# Student Self-Assessment and Faculty Evaluation in Dental Education: A Cross-Sectional Study: Short title: Self-Assessment in Dental Education

**DOI:** 10.31661/gmj.v15i.3908

**Published:** 2026-07-14

**Authors:** Najla N. Al-Dabbagh

**Affiliations:** ^1^ Department of Periodontics, King Abdulaziz University, Jeddah, Saudi Arabia

**Keywords:** Dental education, Faculty evaluation, Reflective practice, Self-assessment, Self-regulated learning, Students

## Abstract

**Background:**

Student self-assessment is emphasized in dental education because it promotes reflective practice, self-regulated learning, and clinical competence. However, its educational value depends on students’ engagement and faculty members’ consistent use of self-assessment in feedback and evaluation.

**Materials and Methods:**

This cross-sectional survey assessed perceptions and practices related to self-assessment among 108 dental students and 46 faculty members. Structured questionnaires collected demographic data, student self-assessment practices, faculty attention to self-assessment forms, perceived influence on final evaluation, and student-reported barriers. Data were analyzed descriptively using frequencies and percentages.

**Results:**

Among students, 31 (28.7%) reported always completing self-assessment forms, 58 (53.7%) completed them frequently, and 19 (17.6%) completed them rarely. Among faculty members, 18 (39.1%) always paid attention to students’ self-assessment forms, 11 (23.9%) frequently did so, 12 (26.1%) occasionally did so, and five (10.9%) rarely did so. Sixty students (55.6%) and 19 faculty members (41.3%) agreed or strongly agreed that self-assessment influenced final evaluation. The most frequently reported barrier was lack of time, reported by 34 students (31.5%).

**Conclusions:**

Self-assessment was commonly used and generally valued, but implementation was inconsistent. Lack of time and uncertainty regarding faculty consideration were important barriers. Clearer integration into clinical assessment is recommended.

## Introduction

Self-assessment is an important component of contemporary health professions education because it encourages learners to evaluate their own performance, identify gaps in competence, and plan further learning [1, 2]. In dental education, students must acquire clinical judgment, technical skill, professionalism, and patient-centered decision-making. Self-assessment can support these outcomes by helping students compare their performance with explicit clinical and educational standards.

Reflective practice is particularly relevant in clinical dental training.

Dental students are expected to evaluate diagnostic reasoning, treatment planning, procedural quality, communication, infection control, and professional behavior. When structured appropriately, self-assessment encourages learners to consider what was performed well, what requires improvement, and how future clinical performance can be enhanced. This process is closely aligned with self-regulated learning, in which students set goals, monitor progress, seek feedback, and modify learning strategies [3, 4].

However, self-assessment is not inherently accurate or effective when used in isolation. Learners may overestimate or underestimate their own performance, particularly when assessment criteria are unclear or when faculty feedback is limited [5, 6]. Faculty evaluation and feedback therefore remain central to the educational value of self-assessment. Faculty members help students interpret criteria, calibrate judgments, and translate reflection into specific improvement plans. Accordingly, self-assessment should complement, rather than replace, faculty evaluation.

A persistent challenge in clinical education is the gap between student self-assessment and faculty evaluation. Students may perceive self-assessment as meaningful only when they believe that faculty members review and consider it. Conversely, faculty members may support self-assessment conceptually but apply it inconsistently because of workload, limited time, absence of standardized rubrics, or uncertainty about how self-assessment should contribute to final grades [7, 8]. These inconsistencies may reduce student motivation and limit the role of self-assessment in reflective learning. Institution-specific evidence remains important because curriculum structures, assessment systems, and faculty practices vary across dental schools. Understanding both student and faculty perspectives can identify practical barriers and guide improvements in clinical assessment design. Therefore, this study aimed to assess dental students’ self-assessment practices, faculty attention to student self-assessment forms, perceived influence on final evaluation, and student-reported barriers to completing self-assessment in a dental education setting.

## Materials and Methods

### Study Design and Setting

This cross-sectional survey study assessed student and faculty perceptions and practices related to self-assessment in dental education. Cross-sectional designs are appropriate for describing attitudes, behaviors, and perceptions at a defined point in time [9]. The study was conducted at king Abdulaziz university faculty of dentistry, Jeddah, Saudi Arabia, within the clinical dental education setting.

### Participants

The study included two participant groups: dental students and faculty members. A total of 108 dental students and 46 faculty members completed the questionnaires. Student participants were enrolled in the fourth, fifth, or sixth year of the dental program. Faculty participants were involved in dental education and clinical evaluation. Participants were recruited using a survey-based approach during the study period. Eligibility criteria, exclusion criteria, total number of invited participants, and response rate should be inserted if available.

### Questionnaire Development

Two structured questionnaires were used: one for dental students and one for faculty members. The student questionnaire collected demographic information, frequency of completing self-assessment forms, perceived influence of self-assessment on final evaluation, and barriers to completing self-assessment. The faculty questionnaire collected demographic information, frequency of attention to student self-assessment forms, perceptions of the importance of student self-assessment, and perceived influence on final grades. Questionnaire validation and pilot testing should be reported. If content validation was performed, the number and expertise of reviewers, item revision process, and content validity procedures should be described. If pilot testing was performed, the pilot sample, clarity assessment, completion time, and internal consistency results should be reported. If no validation or pilot testing was conducted, this should be acknowledged as a limitation. Data Collection Data were collected using structured questionnaires administered to students and faculty members during the study period. Participation was voluntary. The questionnaire did not collect identifying information, and responses were analyzed anonymously. Completed questionnaires were reviewed for completeness before analysis.

### Ethical Approval

This study was approved by the Institutional Review Board of king Abdulaziz university faculty of dentistry under approval number 172-11-25. The study was conducted in accordance with the ethical principles of the Declaration of Helsinki. Informed consent was obtained from all participants before questionnaire completion. Participation was voluntary, and responses were collected anonymously.

### Statistical Analysis

Data were analyzed using IBM SPSS Statistics for Windows, version 26.0 (IBM Corp., Armonk, NY, USA) . Categorical variables were summarized as frequencies and percentages. No inferential statistics were performed because no P-values were provided. If inferential analysis is later added, associations between student and faculty responses should be assessed using the chi-square test or Fisher’s exact test, as appropriate, and exact P-values should be reported.

## Results

**Table:** Table1. Participant Demographic Characteristics

Characteristic	Students (n=108), n (%)	Faculty members (n=46), n (%)
Gender		
Male	46 (42.6)	16 (34.8)
Female	62 (57.4)	30 (65.2)
Age group		
20–24 years	96 (88.9)	Not applicable
25 years or older	12 (11.1)	Not applicable
25–34 years	Not applicable	2 (4.3)
35–44 years	Not applicable	33 (71.7)
45–54 years	Not applicable	7 (15.2)
55–64 years	Not applicable	4 (8.7)
Academic year		
Fourth year	44 (40.7)	Not applicable
Fifth year	16 (14.8)	Not applicable
Sixth year	48 (44.4)	Not applicable
Faculty working experience		
One–five years	Not applicable	7 (15.2)
Five–10 years	Not applicable	18 (39.1)
10–20 years	Not applicable	14 (30.4)
More than 20 years	Not applicable	7 (15.2)

Note: Values are presented as n (%). Percentages may not total 100 because of rounding.

**Table:** Table2. Self-Assessment Completion and Faculty Attention

Response category	Students completing self-assessment forms (n=108), n (%)	Faculty attention to student self-assessment forms (n=46), n (%)
Always	31 (28.7)	18 (39.1)
Frequently	58 (53.7)	11 (23.9)
Occasionally	Not applicable	12 (26.1)
Rarely	19 (17.6)	5 (10.9)
Never	Not applicable	Not applicable

Note: Values are presented as n (%). “Not applicable” indicates that the response category was not available or not reported for that participant group in the available dataset.

**Table:** Table3. Perceived Influence on Final Evaluation

Response category	Students (n=108), n (%)	Faculty members (n=46), n (%)
Strongly agree	19 (17.6)	6 (13.0)
Agree	41 (38.0)	13 (28.3)
Neutral	32 (29.6)	10 (21.7)
Disagree	10 (9.3)	14 (30.4)
Strongly disagree	6 (5.6)	3 (6.5)

Note: Values are presented as n (%). Percentages may not total 100 because of rounding.

**Table:** Table4. Student-Reported Barriers

Barrier	Students (n=108), n (%)
Lack of time	34 (31.5)
Doubt that faculty members consider self-assessment	12 (11.1)
Perceived irrelevance to final evaluation	11 (10.2)
Perception of predetermined grading	9 (8.3)
Forgetfulness	8 (7.4)
Other reasons	34 (31.5)

Note: Values are presented as n (%). The “Other reasons” category should be specified if open-text responses are available.

A total of 108 dental students and 46 faculty members were included in the analysis. Among students, 62 (57.4%) were female and 46 (42.6%) were male.

Most students were aged 20–24 years (n=96, 88.9%), and 12 (11.1%) were aged 25 years or older. Regarding academic year, 44 students (40.7%) were in the fourth year, 16 (14.8%) were in the fifth year, and 48 (44.4%) were in the sixth year.

Among faculty members, 30 (65.2%) were female and 16 (34.8%) were male. Faculty age distribution was as follows: two (4.3%) were aged 25–34 years, 33 (71.7%) were aged 35–44 years, seven (15.2%) were aged 45–54 years, and four (8.7%) were aged 55–64 years. Faculty working experience was most commonly five–10 years (n=18, 39.1%), followed by 10–20 years (n=14, 30.4%), one–five years (n=7, 15.2%), and more than 20 years (n=7, 15.2%) (Table1).

Among students, 31 (28.7%) reported always completing self-assessment forms, 58 (53.7%) completed them frequently, and 19 (17.6%) completed them rarely. Among faculty members, 18 (39.1%) reported always paying attention to students’ self-assessment forms, 11 (23.9%) frequently did so, 12 (26.1%) occasionally did so, and five (10.9%) rarely did so (Table2).

In students, 19 (17.6%) strongly agreed and 41 (38.0%) agreed that self-assessment influenced final evaluation. Thirty-two students (29.6%) were neutral, 10 (9.3%) disagreed, and six (5.6%) strongly disagreed.

In faculty members, six (13.0%) strongly agreed and 13 (28.3%) agreed that self-assessment influenced final grades. Ten faculty members (21.7%) were neutral, 14 (30.4%) disagreed, and three (6.5%) strongly disagreed (Table3).

Faculty members were also asked about the importance of student evaluation of their clinical cases. Twenty faculty members (43.5%) strongly agreed, 17 (37.0%) agreed, six (13.0%) were neutral, two (4.3%) disagreed, and one (2.2%) strongly disagreed.

The most frequently reported barrier to completing self-assessment forms was lack of time, reported by 34 students (31.5%). Twelve students (11.1%) reported doubt that faculty members would consider their self-assessments, and 11 (10.2%) perceived self-assessment as irrelevant to final evaluation. Nine students (8.3%) reported a perception of predetermined grading by faculty, and eight (7.4%) reported forgetfulness. Thirty-four students (31.5%) selected other reasons (Table4).

No inferential statistics were performed.

## Discussion

This cross-sectional study examined student self-assessment practices and faculty evaluation perspectives in a dental education setting. The main findings indicate that self-assessment was commonly used by students, with more than four-fifths reporting that they completed self-assessment forms always or frequently. Faculty members also reported attention to student self-assessment forms, although the frequency of attention varied. These findings suggest that self-assessment is present within the assessment culture of the program but may not be implemented consistently across clinical settings.

Self-assessment is valuable in dental and health professions education because it supports reflective practice and encourages learners to participate actively in their development [1, 2]. In clinical dental training, students must learn not only to perform procedures but also to judge the quality of their own work against professional standards. The present findings show that many students reported regular engagement with self-assessment. However, frequent completion alone does not ensure meaningful reflection. Self-assessment is most effective when students use clear criteria, receive timely feedback, and have opportunities to modify subsequent performance [3, 4]. The results also demonstrate that student and faculty perceptions were not fully aligned regarding the influence of self-assessment on final evaluation. More than half of the students agreed or strongly agreed that self-assessment influenced final evaluation, whereas fewer than half of faculty members agreed or strongly agreed. In addition, a notable proportion of faculty members disagreed that self-assessment influenced final grades. This finding suggests uncertainty about whether self-assessment is primarily formative, summative, or both. Such ambiguity may weaken students’ trust in the assessment process and reduce the motivational value of self-assessment.

Previous literature has cautioned that self-assessment should not be interpreted as an inherently accurate substitute for expert judgment [5, 6]. Students may lack the experience needed to evaluate clinical performance accurately, particularly when criteria are implicit or faculty expectations vary. Therefore, self-assessment should be used within a broader assessment system that includes faculty evaluation, structured rubrics, feedback conversations, and repeated calibration opportunities. Faculty guidance is especially important in helping students develop evaluative judgment, defined as the ability to judge the quality of one’s own work and that of others [10, 11].

The most common student-reported barrier was lack of time. This finding is educationally important because dental curricula are often clinically intensive, and students may perceive self-assessment as an additional administrative task rather than a learning activity. To address this barrier, self-assessment should be incorporated into scheduled clinical workflows rather than added after clinical requirements are completed. Protected reflection time, short structured prompts, and integration of self-assessment into case discussions may improve completion and quality.

Another important barrier was uncertainty about whether faculty members considered student self-assessments. This concern is consistent with the observed variation in faculty attention to self-assessment forms. If students believe that faculty members do not review their reflections, self-assessment may become superficial. Faculty members may also vary in attention because of workload, limited time, lack of standardized rubrics, or uncertainty about how self-assessment should influence grading. Faculty calibration sessions may improve consistency by clarifying expectations, defining how self-assessment should be reviewed, and aligning student reflection with clinical competency standards.

The findings have several implications for dental education. First, self-assessment should be explicitly linked to learning outcomes and clinical competencies. Second, structured rubrics should be used to help students compare their performance with defined criteria. Third, faculty members should provide timely feedback that acknowledges student self-assessment and identifies specific improvement strategies. Fourth, institutions should consider faculty development and calibration sessions to ensure consistent interpretation and use of self-assessment forms.

This study has limitations. First, the cross-sectional design captures perceptions at one point in time and cannot determine causal relationships between self-assessment and academic or clinical performance. Second, the study was conducted in a single institution, which may limit generalizability. Third, the study relied on self-reported questionnaire responses, which may be influenced by recall bias or social desirability bias. Fourth, questionnaire validation and pilot testing were not reported in the available manuscript, limiting confidence in the measurement properties of the instrument. Fifth, only descriptive analysis was reported, limiting the ability to determine whether differences between student and faculty responses were statistically significant. Sixth, some categories in the original manuscript and appendix were inconsistent and required correction based on the appendix-supported denominator and response counts.

Future research should use validated instruments, multicenter sampling, and mixed-methods designs to examine how students and faculty interpret self-assessment in clinical dental education. Qualitative interviews or focus groups could provide deeper insight into why students do not complete self-assessment forms and why faculty members may attend to them inconsistently. Longitudinal studies are also needed to determine whether structured self-assessment combined with faculty feedback improves clinical performance, reflective capacity, and self-regulated learning.

## Conclusion

Self-assessment was commonly used and generally valued by students and faculty members in this dental education setting. However, implementation was inconsistent, particularly regarding faculty attention to self-assessment forms and the perceived influence of self-assessment on final evaluation. Lack of time and uncertainty about whether faculty members considered self-assessment were major student-reported barriers. Clearer integration of self-assessment into clinical assessment, structured rubrics, protected reflection time, faculty calibration, and timely feedback are recommended. Further multicenter research using validated tools is needed to evaluate the educational impact of self-assessment in dental education.

## Conflict of Interest

The author declares no conflicts of interest.

## AI Disclosure Statement

During the preparation of this work, the authors used ChatGPT to check grammar, spelling, and the American style of writing for some sentences. After using this tool, the authors reviewed and edited the content as needed, taking full responsibility for the publication's content.
